# FASTMAP—a flexible and scalable immunopeptidomics pipeline for HLA- and antigen-specific T-cell epitope mapping based on artificial antigen-presenting cells

**DOI:** 10.3389/fimmu.2024.1386160

**Published:** 2024-05-08

**Authors:** Luisa Weisbrod, Luigi Capriotti, Marco Hofmann, Valerie Spieler, Herbert Dersch, Bernd Voedisch, Peter Schmidt, Susanne Knake

**Affiliations:** ^1^ Recombinant Protein Discovery, CSL Innovation GmbH, Marburg, Germany; ^2^ Analytical Biochemistry, Research and Development, CSL Behring AG, Bern, Switzerland; ^3^ Protein Biochemistry, Bio21 Institute, CSL Limited, Parkville, VIC, Australia; ^4^ Department of Neurology, Epilepsy Center Hessen, Philipps University Marburg, Marburg, Germany

**Keywords:** T-cell epitope mapping, HLA, antigen-specific, autoimmunity, artificial antigen-presenting cells, immunopeptidomics, immunotherapy

## Abstract

The study of peptide repertoires presented by major histocompatibility complex (MHC) molecules and the identification of potential T-cell epitopes contribute to a multitude of immunopeptidome-based treatment approaches. Epitope mapping is essential for the development of promising epitope-based approaches in vaccination as well as for innovative therapeutics for autoimmune diseases, infectious diseases, and cancer. It also plays a critical role in the immunogenicity assessment of protein therapeutics with regard to safety and efficacy concerns. The main challenge emerges from the highly polymorphic nature of the human leukocyte antigen (HLA) molecules leading to the requirement of a peptide mapping strategy for a single HLA allele. As many autoimmune diseases are linked to at least one specific antigen, we established FASTMAP, an innovative strategy to transiently co-transfect a single HLA allele combined with a disease-specific antigen into a human cell line. This approach allows the specific identification of HLA-bound peptides using liquid chromatography–tandem mass spectrometry (LC-MS/MS). Using FASTMAP, we found a comparable spectrum of endogenous peptides presented by the most frequently expressed HLA alleles in the world’s population compared to what has been described in literature. To ensure a reliable peptide mapping workflow, we combined the HLA alleles with well-known human model antigens like coagulation factor VIII, acetylcholine receptor subunit alpha, protein structures of the SARS-CoV-2 virus, and myelin basic protein. Using these model antigens, we have been able to identify a broad range of peptides that are in line with already published and *in silico* predicted T-cell epitopes of the specific HLA/model antigen combination. The transient co-expression of a single affinity-tagged MHC molecule combined with a disease-specific antigen in a human cell line in our FASTMAP pipeline provides the opportunity to identify potential T-cell epitopes/endogenously processed MHC-bound peptides in a very cost-effective, fast, and customizable system with high-throughput potential.

## 1 Introduction

Autoimmune diseases are estimated to affect approximately 10% of the world’s population, with a significant rise over the last decades (1–4). Various factors increase the risk of people to experience autoimmunity, such as sex (women are three times more affected than men) (5), genetic susceptibility, and a series of environmental triggers. All autoimmune disorders share the failure of the immune system to differentiate between self and non-self, consequently impairing the mechanism of immune tolerance (1).

The critical part of the adaptive immune system to maintain self-tolerance and activate an effective immune response is represented by major histocompatibility molecule classes I and II (MHC-I and MHC-II molecules) that present antigen-specific peptides to CD8+ and CD4+ T cells, respectively. MHC-I molecules consist of a heavy chain (α chain) comprising the transmembrane domain and a non-covalently linked light chain, the β2-microglobulin (β2M). The α chain is composed of three helices, α1–3, in which the peptide-binding groove is located between α1 and α2 (6). MHC-II is an α/β heterodimer of two chains anchored in the membrane by transmembrane helices. Peptides bind in a groove formed by α and β chain, allowing a peptide length of approximately 13 to 25 amino acids compared to shorter peptide sequences of generally 8 to 15 amino acids for MHC-I (in special cases up to 25 amino acids) (7–9).

Most MHC-I molecules bind antigenic peptides derived from the degradation of proteins by the proteasome (10) and carried into the endoplasmic reticulum (ER) via the transporter associated with antigen processing (TAP). Peptides are trimmed by ER aminopeptidases (ERAP) and loaded onto the MHC-I for presentation on the cell surface of almost all nucleated cells (11). A minor part of MHC-I peptide ligands is derived by lysosomal protein degradation or a pathway from endosome to the cytosol (cross-presentation) (12, 13).

While the MHC-I displays peptides from intracellular events such as bacteria, viral infection, or cellular transformation, the MHC-II processing pathway especially encompasses the extracellular milieu (14, 15). MHC-II molecules are predominantly expressed on professional antigen-presenting cells (APCs) such as dendritic cells, B cells, macrophages, and thymic epithelial cells (16). Exogenous proteins are incorporated via phagocytosis and endocytosis; endogenous proteins enter the lysosome MHC class II compartment (MIIC) via autophagy. The MHC-II dimer is assembled in the ER as a complex with the invariant chain (li) that functions as a placeholder for the peptide. The MHC-II complex traffics to the MIIC for further processing of li into a peptide called class II-associated invariant chain peptide (CLIP). The chaperone-like molecules HLA-DM and HLA-DO facilitate the release of CLIP and the binding by a high-affinity peptide or even block the peptide exchange. The peptide-loaded MHC-II molecule is transported to the cell surface to present the peptide (14, 17).

For the development of antigen-specific immunotherapies (ASI) that hold a great promise for a broad range of autoimmune diseases, it is an indispensable step to identify the MHC-bound peptides causing the T cell-mediated immune response. The HLA’s highly polygenic and polymorphic nature, combined with the complexity of autoimmune diseases, pose a major challenge in predicting antigen-specific T-cell epitopes. The result of MHC polymorphism is a variation in the peptide-binding groove that significantly affects the peptide-binding specificity. In addition, expression levels vary between the different HLA genotypes and influence the peptide repertoire (11, 18). In the past few years, *in silico* prediction tools for HLA-binding peptides have significantly improved; however, the accuracy of the prediction depends on the data the algorithm is based on, so a carefully chosen collection of datasets is essential. Nevertheless, a major drawback of computational prediction tools is the high rate of false-positive peptide predictions potentially caused by variable factors influencing the MHC–peptide binding. Important factors are antigen processing (19) and the stability of the peptide/MHC complex (20) that both influence the likelihood of a peptide to be presented. Antigenic peptide editing chaperones and the peptide loading complex (PLC) play a crucial role in antigen processing. Antigenic peptide editing chaperones are a group of proteins involved in the selection and loading of antigenic peptides onto MHC-I molecules that ensure that only high-affinity, properly processed peptides are presented on the cell surface. Calreticulin (CRT) is an ER-resident protein that assists in the selection of high-affinity peptides. It aids in peptide trimming, helping to remove inappropriate or poorly fitting peptides from the MHC-I binding groove. The second main chaperone represents tapasin, another ER-resident protein that plays a critical role in MHC class I peptide selection. It facilitates the loading of peptides by promoting the association of the MHC-I heavy chain with TAP, which transports peptides from the cytosol into the ER. Both chaperones act as quality control mechanism for peptide presentation on MHC-I molecules. The PLC is a multi-protein assembly located in the ER and is responsible for the final steps of MHC-I antigen presentation, where antigenic peptides are loaded onto MHC-I molecules. The key components of the PLC include the MHC-I molecule, TAP, tapasin, calreticulin, ERp57, and calnexin. The latter two help in the formation of stable peptide-loaded MHC-I complexes and ensure proper folding and assembly of the MHC-I molecule. All these factors are important for the selection mechanism of peptide presentation (17, 21–23).

Artificial APCs (aAPCs) represent a powerful tool for the precise identification of MHC-restricted peptides and T-cell epitopes, for investigation of the factors influencing the immunopeptidome and for principles of restoring the immunological tolerance to self-antigens. Furthermore, much effort has been put into the identification of so-called neoepitopes in the context of cancer conceiving the approach of affinity-tagged MHC-I and -II molecules to learn binding rules facilitated by mass spectrometry (24, 25).

A key obstacle of mass spectrometry-based MHC-restricted peptide mapping is the limited effectiveness of antigen processing and presentation pathways (26, 27) and the resulting need of high cell numbers for peptide mapping. In our FASTMAP pipeline, we addressed this bottleneck by combining the expression of a single affinity-tagged HLA allele with a specific antigen related to the corresponding autoimmune disease to facilitate an HLA- and antigen-specific peptide mapping based on mass spectrometry analysis. To demonstrate the reliability and reproducibility of our strategy, we combined HLA alleles with well-known human model antigens like myelin basic protein (MBP), acetylcholine receptor subunit alpha (AChRα), a B-domain-deleted coagulation factor VIII (BDD-rFVIII), and the spike glycoprotein and nucleoprotein of the Wuhan variant of SARS-CoV-2. A large overlap of the peptide spectra was found when the mapped epitopes from our pipeline were compared to peptides gathered in the Immune Epitope Database (IEDB), the prediction tools NetMHCpan-4.1 and NetMHCIIpan-4.0, and the selected literature. In summary, FASTMAP allows, via the transient co-expression of a single HLA allele and antigen in a human cell line, endogenous processing of the peptides in a cost-effective, rapid, flexible, and scalable system with high-throughput potential.

## 2 Materials and methods

### 2.1 Plasmid construction/DNA construct design

Gene sequences for the design of recombinant expression constructs of selected HLA-I and -II alleles were identified by the IPD-IMGT/HLA website (https://www.ebi.ac.uk/ipd/imgt/hla/alleles/). The construct for HLA-I consisted of the α chain C-terminally fused to the Twin-Strep-tag® sequence [SAWSHPQFEKGGGSGGGSGGSAWSHPQFEK (28)]. The HLA-I construct was cloned into an in-house-modified pIRES vector via the BamHI/NotI or NheI/NotI restriction sites. The constructs representing the HLA-II alleles (DR, DP, and DQ) were cloned using the same restriction sites to generate a basic plasmid construct for DR, DP, and DQ. In the case of DR, a fragment consists of the β chain sequence C-terminally linked to the Twin-Strep-tag®, a short GSG-linker, and a P2A self-cleaving peptide (ATNFSLLKQAGDVEENPGP). The α chain is connected to the P2A sequence via an additional GS-linker (GGSGGSGGSGGSGGSG) and C-terminally tagged with an HA-Tag (GSYPYDVPDYA). For allelic variation of the DR genotype, the sequence of HLA-DRA1*01:01 was maintained and the β chain was exchanged by inserting an NgoMIV/NaeI restriction site in the P2A peptide sequence. For variation of DP and DQ alleles, the whole construct including α and β chain was exchanged using BamHI/NotI or NheI/NotI as restriction sites.

Antigen sequences were obtained from UniProt: the Universal Protein Knowledgebase (https://www.uniprot.org/). To facilitate the targeting of the antigens to the HLA-II processing pathway, we fused parts of the transmembrane protein lysosome-associated membrane protein 1 (LAMP1) to the C and N termini of the antigens as described before (29). Briefly, the LAMP1 sorting signal at the N terminus (MAAPGSARRPLLLLLLLLLLGLMHCASA) was combined with the appropriate antigen sequence, a GGSGGS-linker, and the LAMP1 tail sequence (NSMLIPIAVGGALAGLVLIVLIAYLVGRKRSHAGYQTI). The construct was tagged at the C terminus with a Myc-tag (EQKLISEEDL) and a C-tag (EPEA), both linked via GGSGGS sequences. Antigen sequences were cloned into a pcDNA vector using BamHI/NotI or NheI/NotI as restriction sites.

All constructs were human-codon-optimized using GENEius for synthetic gene optimization (Eurofins Genomics, BioLink Informationstechnologie).

### 2.2 HLA-I and -II allele frequencies

Seven HLA-I alleles representing the most abundant alleles for HLA-A, -B, and -C of the world’s population and eight alleles sharing a high frequency of HLA-DR, -DP, and -DQ were selected for recombinant expression. Allele frequencies were obtained from AlleleFrequencies.net database and were downloaded on 25 April 2023 from http://www.allelefrequencies.net/. In detail, we have selected HLA-A*02:01, A*24:02, A*11:01, B*35:01, B*07:02, C*04:01, and C*07:02 as well as HLA-DRB1*07:01, DRB1*15:01, DRB1*04:01, and DRB1*03:01 (all combined with HLA-DRA1*01:01). For DQ, we combined HLA-DQA1*05:01 with DQB1*03:01 and DQA1*01:01 with DQB1*05:01. DP alleles were represented by combinations of HLA-DPA1*03:01 with DPB1*04:01 and, additionally, DPA1*02:04 with DPB1*05:01.

### 2.3 TaqMan™ Array for human antigen processing and presentation by MHCs

The Applied Biosystems™ TaqMan™ Array Human Antigen Processing and Presentation by MHCS (Cat. No. 4414104) was performed according to the manufacturer’s protocol. The 96-well plate contained 44 assays to antigen processing and presentation by MHCS-associated genes and four assays to candidate endogenous control genes. All assays were plated in duplicate. Four groups of MHC-related genes were tested, namely, MHC-I, MHC-II, proteasomes, and tapasin.

### 2.4 HLA genotyping

HLA genotyping was performed by DKMS Life Science Lab gGmbH using extracted DNA of Expi293F cells. Samples were analyzed using Illumina Sequencing by Synthesis (NGS) for genotyping of exons 2 and 3 for HLA-A, HLA-B, HLA-C, HLA-DRB1, HLA-DRB3/4/5, HLA-DQA1, HLA-DQB1, HLA-DPA1, and HLA-DPB1.

### 2.5 Cell culture and transfection conditions

The Expi293™ Expression System (ThermoFisher) was used to generate artificial APCs transiently expressing a single affinity-tagged HLA allele together with a selected antigen. Expi293F™ cells were maintained by passaging them every 3 to 4 days in Expi293™ Expression Medium reaching a density of approximately 3–5 × 10^6^ viable cells/mL. The suspension cells were cultured in an orbital shaker in 250-mL conical flasks at 37°C, 160 rpm, 80% humidity, and 8% CO_2_.

Transfections were performed according to the Expi293™ Expression System User Guide (ThermoFisher) with slight modifications. Briefly, cells were allowed to recover for at least three passages after thawing, reaching a viability ≥95%. On the day prior to transfection, cell suspensions with a density of approximately 3–4 × 10^6^ viable cells/mL were diluted 1:2 with Expi293™ Expression Medium and allowed to grow overnight. On the day of transfection, cells were checked for viability (≥95%) and density (approximately 3–5 × 10^6^ viable cells/mL) and diluted to a final density of 2.5 × 10^6^ viable cells/mL with fresh medium. For the preparation of the ExpiFectamine™293/plasmid DNA complexes, 1 µg of plasmid DNA per milliliter of cell culture was used. In detail, 30 µg of plasmid DNA (24 µg HLA, 6 µg antigen) was diluted with 1.5 mL of Opti-MEM™ I Reduced Serum Medium. ExpiFectamine293™ Reagent (80 µL) was mixed thoroughly with 1.5 mL of Opti-MEM™ I Reduced Serum Medium and incubated at room temperature for 2 to 3 min. Then, the diluted ExpiFectamine293™ Reagent was incubated with the DNA plasmid complexes for 15–20 min, and slowly transferred to the cell solution. The cells were incubated in a shaker as described above. A mixture of ExpiFectamine™ Transfection Enhancer 1 and ExpiFectamine™ Transfection Enhancer 2 (150 µL of Enhancer 1 and 1.5 mL of Enhancer 2) was added to the transfected cells 18–22 h after transfection.

Cell count and viability were determined 72 h after transfection, and the transfected cell suspensions were harvested. For cell harvest, the cell suspensions were centrifuged for 5 min at 300 × *g*. The supernatant was discarded, and the cell pellets were washed twice with DPBS. Prior to the second wash, the pellet was transferred to a 2-mL Eppendorf tube. After the last wash, the supernatant was aspirated, and the cell pellet was frozen and stored at −80°C until further analysis. All samples were collected as three technical replicates.

### 2.6 HLA purification/affinity-tagged HLA–peptide complex isolation

Affinity-tagged HLA–peptide complex isolations were performed from cells expressing Twin-Strep-tagged HLA alleles and negative control cell lines that expressed only endogenous HLA–peptide complexes without Twin-Strep-tag®. Frozen cell pellets containing 5 × 10^7^ cells expressing Twin-Strep-tagged HLA molecules were thawed on ice for 15–20 min. After thawing, cells were lysed by pipetting in 1.2 mL of cold lysis buffer (20 mM Tris-Cl, 100 mM NaCl, 6 mM MgCl_2_, 60 mM n-octyl glucoside, 0.2 mM iodoacetamide, 1 mM EDTA, 1× complete EDTA-free protease inhibitor cocktail, and ≥250 units benzonase nuclease, pH 8) [lysis buffer was modified from Abelin et al. (24)]. Lysates were incubated end-over-end at 4°C on ice for 15 min, mixing the lysate three to four times thoroughly in between. Lysates were centrifuged at 15,000 × *g* at 4°C for 20 min to remove cellular debris and insoluble materials (cleared supernatants were transferred to unused tubes). MagStrep “type3” XT beads (5% suspension, IBA Lifesciences, 2-4090-010) (300 µL per sample) were equilibrated by washing the beads twice with 1 mL of 1× Buffer W (100 mM Tris-Cl, 150 mM NaCl, 1 mM EDTA, and 0.2 mM iodoactemaide, pH 8), placing the suspension in the magnetic separator between the washes to remove the supernatant. Cleared lysate (1.2 mL) containing the MHC/peptide complexes was combined with a volume of magnetic beads corresponding to 300 µL of the initial 5% bead suspension and incubated on an overhead shaker for 2 h at 4°C. Afterwards, the tube was placed in the magnetic separator and the supernatant was removed carefully. The magnet was removed. The beads containing bound HLA were washed twice with 1 mL 1× Buffer W, followed by a second cleaning step washing four times with 1 mL 10 mM Tris (pH 8) and immediately proceeding with the elution of the HLA-bound peptides. The protocol for the purification of Twin-Strep-tag® fusion proteins was modified from Schmidt et al. (28).

### 2.7 HLA-bound peptide elution

Five hundred microliters of 0.1% formic acid was added to the HLA-bound beads and incubated on the overhead shaker for 10 min at room temperature. In the meantime, 10-kDa MWCO filter units (Sartorius, VN01H02) were prepared by rinsing them twice with 500 µL of water and once with 0.1% formic acid. The filter units were placed into a fresh collection tube, and the HLA/peptide/bead suspension in 0.1% formic acid was added and centrifuged at 14,000 × *g* for 10 min. The elution step using 500 µL of 0.1% formic acid was performed a second time including the filtration step. The purified peptides present in the filtrate were collected in a separate tube as peptide fraction after each filtration step. After performing the elution step twice, the filter was rinsed with 150 µL of 0.1% formic acid at 14,000 × *g* for 15 min. The filtrate was added to the purified peptide solution.

The concentrated protein fraction containing the Twin-Strep-tag® purified MHCs was recovered by reverse spinning of the concentrate into a fresh collection tube. In this procedure, the filtrate tube was removed, and the concentrator body was inverted into a new filtrate tube and then spun at up to 2,500 × *g* for 2 min (or pulse for 20–30 s). The purified peptide fraction and the MHC fraction were stored at −80°C.

### 2.8 Sample preparation for mass spectrometry

Frozen peptide samples were evaporated using the SpeedVac system at 45°C. The peptides were resuspended in 23 µL of 0.1% formic acid containing iRT peptide mix (Biognosys AG) for quality assurance of the system by vortexing the solution. The peptide samples were loaded onto Evotips for the Evosep ONE LC instrument (Evosep Biosystems) following the manufacturer’s instructions.

### 2.9 Liquid chromatography and mass spectrometry

Samples were analyzed with an Orbitrap Exploris 240 mass spectrometer (ThermoFisher Scientific) connected to an Evosep ONE LC instrument (Evosep Biosystems). The standard “extended method” (88-min gradient) method was used with a 15-cm column (100 μm ID/3 μm bead size) fitted with a 30-μm ID stainless steel emitter. The mass spectrometer was operated in positive ion mode using the following MS parameters:

The spray voltage was set at 2.1 kV, RF lenses were set at 70%, and the capillary temperature was set at 280°C. For MS1, the resolution was set at 120,000, with a scan range of 350–1400 m/z, and the AGC target was set at 300% with maximum injection time set to AUTO. The subsequent MS2 scan resolution was set to 30,000, and the AGC target was set at 200% with an injection time set to AUTO. The intensity threshold for precursor selection was set to 5.0e4. Unassigned +1 and >+6 charged precursors were excluded.

### 2.10 Data processing

Raw data analysis was performed using Proteome Discoverer v2.5.0.400 (ThermoFisher Scientific) using SEQUEST HT with INFERYS rescoring algorithm workflow (in this specific workflow, the INFERYS node is set between the SEQUEST and percolator node) (30, 31).

The SEQUEST node was searched against the Human SwissProt database (Tax ID:9606) and the antigens used in our experiment. The digestion setting was set to No-Enzyme (unspecific); precursor mass tolerance was set to 10 ppm, and fragment mass tolerance for MS2 spectra was set to 0.02 Da. Oxidation of methionine was set as variable modification and carbamidomethylating of cysteine as fixed modification. The percolator node was set to 1% FDR (false discovery rate) for high-confidence peptides and 5% FDR for medium confidence.

### 2.11 Interpretation of LC-MS/MS data

All samples were prepared as a set of 16 samples, including a sample containing only lysis buffer as vehicle control, a negative control consisting of the lysate of wild-type Expi293F cells, and a positive control made of the lysate of wild-type Expi293F cells and spiked with 10 ng of recombinant human MHC-I allele (HLA-A*02:01) fused to a Twin-Strep-tag® (MHC I-Strep) refolded with SARS-CoV-2 peptide (YLQPRTFLL) and HLA-B*07:02 refolded with CMV pp65 peptide (TPRVTGGGAM) (IBA Lifesciences). The remaining 13 samples were represented by aAPCs expressing various HLA/antigen combinations. Sample sets passed the quality control by identification of the two control peptides, YLQPRTFLL and TPRVTGGGAM, as well as by identifying the antigen of interest only in the appropriate samples transfected with the antigen.

Peptides were filtered for their origin of our model antigens and peptide spectra matches (PSM) were quality-checked manually. Only high-confidence and unambiguous peptides were used for further analysis. If peptides were identified also in the negative control—represented by lysate of cells transiently expressing the relevant antigen without co-expression of a recombinant MHC molecule—they and the corresponding nested set/core were also removed from further analysis.

### 2.12 Clustering mapped peptides into nested sets/consensus sequences/cores

Antigen-related peptide data of each sample were aligned with the antigen sequence using the multiple sequence alignment tool “Clustal Omega” (32). Nested sets/cores of peptides were clustered to identify the consensus sequence described as amino acids shared by all peptides of a core. A consensus sequence from a nested set had to consist of a minimum of two overlapping peptides. Peptide cores that were identified just once in all the samples were excluded from consensus sequence identification.

### 2.13 Sequence properties of MS-identified peptides compared to IEDB and NetMHCpan-4.1/NetMHCIIpan-4.0

Identified consensus sequences (cores) of mapped peptides for each antigen/HLA combination were compared to epitopes listed in IEDB (www.iedb.org) (33) and to prediction results of NetMHCpan-4.1 and NetMHCIIpan-4.0 (https://services.healthtech.dtu.dk/services/NetMHCpan-4.1/ and https://services.healthtech.dtu.dk/services/NetMHCIIpan-4.0/) (34, 35). For the IEDB database search, the parameters were set like the following (exemplary for BDD-rFVIII): organism: *Homo sapiens* (human) (ID:9606), antigen: e.g., Coagulation factor VIII (UniProt: P00451), include positive assays, no B-cell assays (just T cell and MHC ligand), MHC restriction type: class I and II (separately), host: *Homo sapiens* (human). For *in silico* prediction of HLA-I epitopes, the antigen sequence was pasted and combined with the appropriate HLA allele in the software. Peptides were predicted for the length of 8 to 14 amino acids. As the model is based on artificial neural networks and is trained on a combination of peptide sets generated from quantitative binding affinity (BA) and mass spectrometry eluted ligands (EL), we included both BA and EL predictions in our comparison. The threshold for strong binding peptides (SB) was set to 0.500 (% Rank), and for weak binding peptides (WB), it was set to 2.000 (% Rank). The parameters for HLA-II epitope prediction were adapted to cover a peptide length from 9 up to 32 amino acids; the threshold was set to 1.000 (% Rank) for SB and 5.000 (% Rank) for WB. The prediction results were collected in a list for each antigen separated by HLA-I and -II, represented by the %Rank_EL, the BindLevel (noB, WB, and SB), and the BA-affinity (nM). The list can be found in the supplement (**Supplementary Table 1**).

## 3 Results

### 3.1 Creating artificial antigen-presenting cells for HLA- and antigen-specific T-cell epitope mapping

We have selected the Expi293F cell line to facilitate an effective transient co-expression of the HLA molecule and the antigen. To address the question whether the Expi293F cells can process and present HLA-I- and especially HLA-II-related peptides, we performed a TaqMan™ Array for Human Antigen Processing and Presentation by MHCs considering gene expression of MHC-I, MHC-II, proteasomes, and TAP-associated glycoprotein (tapasin)-related genes. Additionally, we performed flow cytometry analyses to confirm the surface expression of endogenous HLA-I and -II molecules as well as for the overexpression of the recombinantly expressed MHC molecules. An intracellular staining for the expression HLA-DM and CD74 indicated the presence of both proteins at a low concentration level (**Supplementary Table 2**). In case of CD74, the low detection level may be linked to the expression of a different isoform of CD74 in Expi293F cells (36). The results underlined the ability of the cell line to process peptides for HLA-I and -II pathway-related presentation. Furthermore, the endogenous HLA genotype of Expi293F cells was determined to be HLA-A*02:01, A*03:01, B*07:02, and C*07:02 for HLA-I and DRB1*15:01, DRB5*01:01, DQA1*01:02, DQB1*06:02, DPA1*01:03, and DPB1*04:02 for HLA-II.

**Figure 1:**
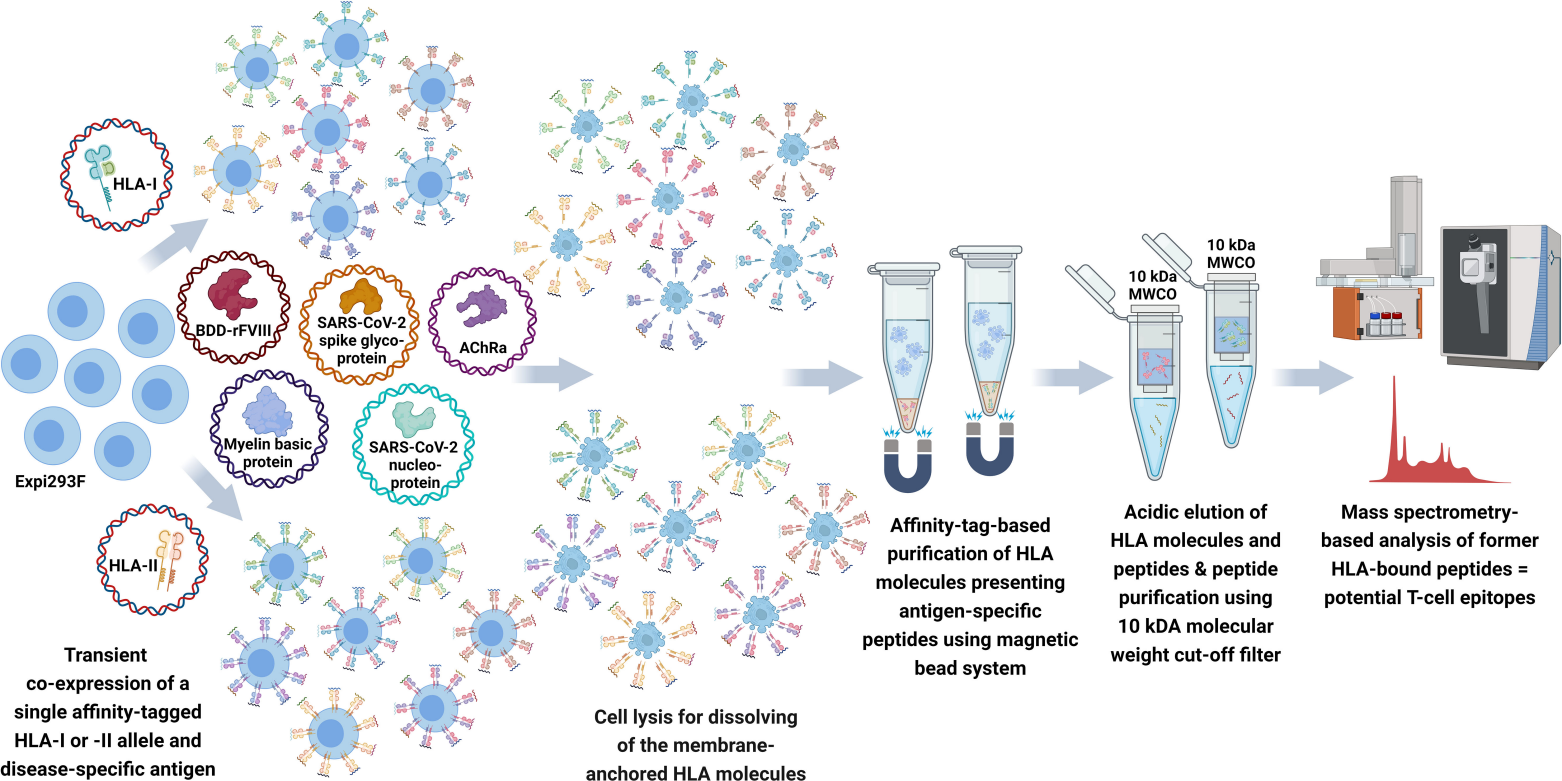
Schematic workflow of FASTMAP for antigen-specific peptide mapping of a single HLA allele. The human cell line Expi293F is transfected with a disease-specific antigen targeting the lysosomal degradation pathway and a single affinity-tagged HLA allele for transient co-expression of both molecules. The recombinant aAPC system shows an overexpression and further presentation of antigen-derived peptides on the surface of the recombinant MHC molecules. MHC molecules equipped with a Twin-Strep-tag® are purified from the Expi293F cell lysate using StrepTactin^®^-coated magnetic beads following excessive washing steps to remove the non-ionic detergent of the lysis buffer. Peptides are eluted by acidic elution and analyzed by nano-flow LC-MS/MS. Created with BioRender.com.

To establish our recombinant aAPC system, we have selected six HLA-I and eight HLA-II alleles covering a high percentage of the prevalent HLA genotypes in the world’s population and considering predispositions for the diseases related to our model antigens. As a proof of concept of our T-cell epitope mapping pipeline, we have chosen model antigens with epitopes published in IEDB and in the literature. Model antigens functioned as SARS-CoV-2 spike glycoprotein (UniProt: P0DTC2) and SARS-CoV-2 nucleocapsid protein (UniProt: P0DTC9) related to a COVID-19 infection, MBP (UniProt: P02686) for multiple sclerosis, and AChRα (UniProt: P02708) for myasthenia gravis. In addition, we included the B-domain-deleted recombinant FVIII (BDD-rFVIII) (Afstyla®, CSL Behring GmbH) related to hemophilia A taking into consideration the detailed data described in Diego et al. and Jankowski et al. (37, 38). To cover the *in silico* prediction tools as a frequently used pipeline with great potential in epitope prediction, we compared our mapped peptides for the model antigens not only with IEDB and literature but also with NetMHCpan-4.1 and NetMHCIIpan-4.0 prediction results. A schematic overview of the FASTMAP workflow is compiled in **Figure 1**.

### 3.2 Establishing FASTMAP as a reproducible pipeline to map T-cell epitopes

An important quality attribute in a highly dynamic process such as antigen processing and presentation combined with a purification workflow and mass spectrometry is the robustness of the process. All HLA/antigen combinations were performed in three technical replicates. Furthermore, we included two positive controls [recombinant human MHC-I allele (HLA-A*02:01) fused to a Twin-Strep-tag® (MHC I-Strep) refolded with SARS-CoV-2 peptide (YLQPRTFLL) and HLA-B*07:02 refolded with CMV pp65 peptide (TPRVTGGGAM)] by spiking them into the cell lysate at the beginning of the purification workflow.

After 72 h of transient expression, peptides were harvested and analyzed as described above. Only high-confidence and unambiguous peptides were used for further analysis.

**Figure 2:**
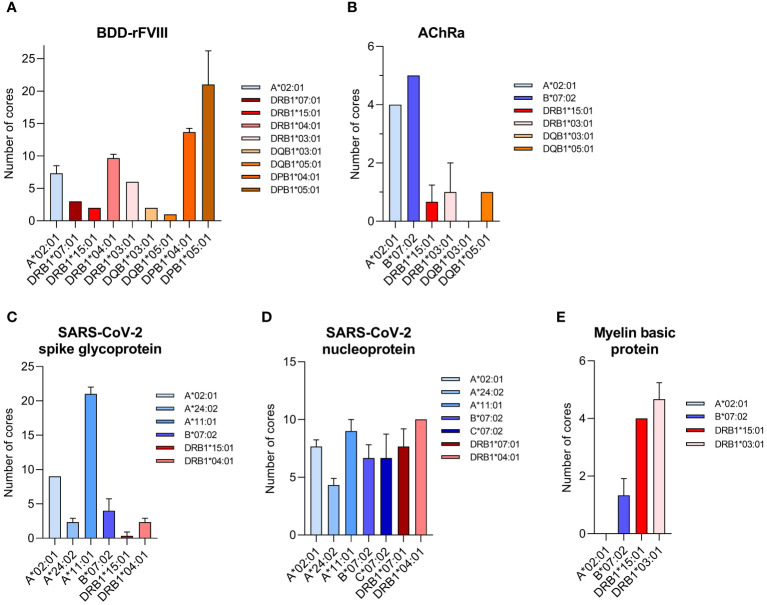
Number of core sequences per individual HLA allele mapped for a specific model antigen. Core sequences are defined by the presence of at least two overlapping peptides containing a conserved core motif, identified as the consensus sequence for the amino acids shared by all peptides of the core. The results are shown for the antigens **(A)** BDD-rFVIII, **(B)** AChRα, **(C)** SARS-CoV-2 spike glycoprotein, **(D)** SARS-CoV-2 nucleoprotein, and **(E)** myelin basic protein. Blue color spectra represent HLA-I alleles, and red color spectra denote HLA-II alleles. Plotted are the mean values of each sample set measured as triplicate. Error bars indicate the standard deviation.

Peptide analysis was performed by multiple sequence alignments (Clustal Omega) of the peptides mapped for each sample. Datasets of each sample measured as triplicate were pooled after alignment of the peptides to the model antigen protein sequence. Nested sets were created sharing at least two overlapping peptides to identify the core/consensus sequence of the peptide set. A representative for various HLA-I alleles combined with SARS-CoV-2 spike glycoprotein and that for various HLA-II alleles in combination with a BDD-rFVIII, respectively, are shown in **Tables 1**–**4**. Finally, we were able to identify up to 38 cores for the five model antigens BDD-rFVIII (9 cores), AChRα (8 cores), SARS-CoV-2 spike glycoprotein (38 cores) and nucleoprotein (15 cores), and MBP (2 cores) in individual combinations with six different HLA-I alleles. For HLA-II-related peptide mapping, we identified up to 47 cores for combinations with eight HLA-II alleles, including BDD-rFVIII (47 cores), AChRα (2 cores), SARS-CoV-2 spike glycoprotein (4 cores) and nucleoprotein (13 cores), and MBP (6 cores). The number of cores identified for each antigen combined with an individual selection of single HLA alleles is shown in **Figure 2**. Plotted are the means of the core number of peptides mapped for each sample set (triplicates), and the error bars indicate the standard deviation (SD). The low SD of the core numbers points out the high reproducibility of our pipeline.

**Table 1:** Representative demonstration of mapped peptides for SARS-CoV-2 spike glycoprotein (UniProt accession P0DTC2) for selected HLA-I alleles HLA-A*02:01, A*24:02, A*11:01, and B*07:02 clustered into cores for identification of the consensus sequence.

Consensus sequence	Start position	End position	Core no.	No. of peptides	Peptide length (amino acids)	HLA types
GVYYPDKVFR	35	44	1	34	10	HLA-A*11:01
GVYFASTEK	89	97	2	8	9	HLA-A*11:01
TLDSKTQSL	109	117	3	8	9	HLA-A*02:01
FLMDLEGKQGNFKNL	175	189	4	11	15	HLA-A*02:01
FVFKNIDGYFK	192	202	5	8	11	HLA-A*11:01
TPINLVRDL	208	216	6	6	9	HLA-B*07:02
YLQPRTFLL	269	277	7	6	9	HLA-A*02:01
TLKSFTVEK	302	310	8	13	9	HLA-A*11:01
FTVEKGIYQT	306	315	9	3	10	HLA-A*02:01
QPTESIVRF	321	329	10	3	9	HLA-B*07:02
ASVYAWNRKR	348	357	11	70	10	HLA-A*11:01
VYAWNRKRI	350	358	12	6	9	HLA-A*24:02
NSASFSTFK	370	378	13	10	9	HLA-A*11:01
KCYGVSPTKL	378	387	14	5	10	HLA-A*11:01
KIADYNYKL	417	425	15	5	9	HLA-A*02:01
KLPDDFTGC	424	432	16	8	9	HLA-A*02:01
NYNYLYRLF	448	456	17	9	9	HLA-A*24:02
VVLSFELLH	511	519	18	3	9	HLA-A*11:01
GTGVLTESNKK	548	558	19	14	11	HLA-A*11:01
AVRDPQTLEIL	575	585	20	2	11	HLA-B*07:02
RVYSTGSNVFQTR	634	646	21	6	13	HLA-A*11:01, HLA-B*07:02
ASYQTQTNSPR	672	682	22	3	11	HLA-A*11:01
GSFCTQLNR	757	765	23	32	9	HLA-A*11:01
RSFIEDLLFNK	815	825	24	5	11	HLA-A*11:01
VTLADAGFIK	826	835	25	4	10	HLA-A*11:01
VTQNVLYENQK	911	921	26	2	11	HLA-A*11:01
NQFNSAIGK	925	933	27	2	9	HLA-A*11:01
SLSSTASALGK	937	947	28	4	11	HLA-A*11:01
AQALNTLVK	956	964	29	23	9	HLA-A*11:01
ALNTLVKQL	958	965	30	4	9	HLA-A*02:01
SVLNDILSRLDK	975	986	31	44	12	HLA-A*11:01
VLNDILSRL	976	984	32	6	9	HLA-A*02:01
RLITGRLQSL	995	1,004	33	6	10	HLA-A*02:01, HLA-B*07:02
RAAEIRASANL	1,014	1,024	34	3	11	HLA-B*07:02
ASANLAATK	1,020	1,028	35	3	9	HLA-A*11:01
VTYVPAQEK	1,065	1,073	36	4	9	HLA-A*11:01
GTHWFVTQR	1,099	1,107	37	105	9	HLA-A*11:01
QYIKWPWYI	1,208	1,216	38	2	9	HLA-A*24:02

Consensus sequences are underlined and defined as the series of amino acids that are shared by all peptides belonging to the nested sets. Nested sets of at least two overlapping peptides are created. Data shown are pooled from three technical replicates.

**Table 2:** Exemplary presentation of the individual peptides mapped for SARS-CoV-2 spike glycoprotein (UniProt accession P0DTC2) for HLA-I allele HLA-A*02:01 (14.1-14.3) that are included in the nested set for peptide “YLQPRTFLL”.

Sample no.	Peptide									Core no.
14.1	Y	L	Q	P	R	T	F	L	L	7
	Y	L	Q	P	R	T	F	L	L	
14.2	Y	L	Q	P	R	T	F	L	L	
	Y	L	Q	P	R	T	F	L	L	
14.3	Y	L	Q	P	R	T	F	L	L	
	Y	L	Q	P	R	T	F	L	L	

Nested sets of at least two overlapping peptides are created. Sample no. represents the peptides mapped for this set for each technical replicate.

**Table 3:** Representative demonstration of mapped peptides for human coagulation factor FVIII (UniProt accession P00451) for selected HLA-II alleles HLA-DRB1*07:01, DRB1*15:01, DRB1*04:01, DRB1*03:01, DQA1*05:01 & DQB1*03:01, DQA1*01:01& DQB1*05:01, DPA1*01:03 & DPB1*04:01, and DPA1*02:04 & DPB1*05:01 clustered into cores for identification of the consensus sequence.

Consensus sequence	Start position	End position	Core no.	No. of peptides	Peptide length (amino acids)	HLA types
TSVVYKKTLFVEFTDHLFN	61	79	1	243	19	HLA-DPB1*04:01, HLA-DPB1*05:01
KKTLFVEFTDHLFNIA	66	81	2	23	16	HLA-DQB1*05:01, HLA-DPB1*04:01, HLA-DPB1*05:01
GPTIQAEVYDTVVITLKNMASHPVS	92	116	3	14	25	HLA-DPB1*05:01
VYDTVVITLKNMASHPVSLHA	99	119	4	23	21	HLA-DRB1*04:01
LTYSYLSHVDLVKDLNSGLIG	173	193	5	5	21	HLA-DPB1*05:01
KEKTQTLHKFILLFAVFDEGKSWH	205	228	6	16	24	HLA-DPB1*05:01
SETKNSLMQDRDAASARAWPK	229	249	7	183	21	HLA-DRB1*04:01, HLA-DRB1*03:01
IGCHRKSVYWHVIGMG	265	280	8	6	16	HLA-DPB1*05:01
DSEMDVVRFDDDNSPSFIQIRSVAKKHPKT	371	400	9	289	30	HLA-DRB1*07:01, HLA-DRB1*04:01
SVAKKHPKTWVHYIAAEEEDWD	392	413	10	181	22	HLA-DPB1*05:01
YIAAEEEDWDYAPLVLAPDDRSYKSQYLN	404	432	11	76	29	HLA-DRB1*03:01
IGRKYKKVRFMAYTDET	438	454	12	7	17	HLA-DPB1*04:01
GPLLYGEVGDTLLIIFKNQASRPYN	469	493	13	103	25	HLA-DRB1*15:01, HLA-DRB1*04:01
IYPHGITDVRPLYSRRLPK	494	512	14	11	19	HLA-DRB1*03:01
SDPRCLTRYYSSFVNMERDLA	543	563	15	13	21	HLA-DPB1*05:01
RGNQIMSDKRNVILFSV	581	597	16	21	17	HLA-DRB1*03:01
ESVDQRGNQIMSDKRNVILFSVFDENRS	576	603	17	113	28	HLA-DPB1*05:01
RSWYLTENIQRFLPNPA	602	618	18	3	17	HLA-DPB1*05:01
IGAQTDFLSVFFSGYTFKHKMV	661	682	19	55	22	HLA-DPB1*04:01, HLA-DPB1*05:01
NSDFRNRGMTALLKVSSCDKN	713	733	20	44	21	HLA-DPB1*05:01
EDSYEDISAYLLSKNNAIEPRS	739	760	21	112	22	HLA-DRB1*07:01, HLA-DRB1*04:01
SPRSFQKKTRHYFIAAVERLW	1,706	1,726	22	149	21	HLA-DPB1*05:01
ERLWDYGMSSSPHVLRNR	1,723	1,740	23	5	18	HLA-DRB1*07:01
NRAQSGSVPQFKKVVFQEFTDGSFTQPLYRGELN	1,739	1,772	24	181	34	HLA-DPB1*04:01, HLA-DPB1*05:01
AEVEDNIMVTFRNQASRPYSF	1,784	1,804	25	21	21	HLA-DRB1*15:01, HLA-DRB1*04:01
FRNQASRPYSFYSSLISYEEDQRQGAEPRKN	1,794	1,824	26	40	31	HLA-DPB1*05:01
SSLISYEEDQRQGAEPR	1,806	1,822	27	5	17	HLA-DRB1*04:01
TYFWKVQHHMAPTKDE	1,833	1,848	28	5	16	HLA-DPB1*05:01
SDVDLEKDVHSGLIGP	1,858	1,873	29	15	16	HLA-DRB1*03:01, HLA-DQB1*03:01
TLNPAHGRQVTVQEFALF	1,881	1,898	30	63	18	HLA-DPB1*04:01
ENIHSIHFSGH	1,970	1,980	31	2	11	HLA-DPB1*04:01
FSGHVFTVRKKEEYK	1,977	1,991	32	4	15	HLA-DPB1*05:01
TVRKKEEYKMALYNLYPGVFE	1,983	2,003	33	239	21	HLA-DPB1*04:01, HLA-DPB1*05:01
LYNLYPGVFETVEMLPSKAGIWR	1,994	2,016	34	25	23	HLA-DPB1*04:01, HLA-DPB1*05:01
HAGMSTLFLVYSNK	2,026	2,039	35	10	14	HLA-DPB1*04:01
APKLARLHYSGSINAWSTKEP	2,066	2,086	36	7	21	HLA-DRB1*04:01
WSTKEPFSWIKVDLLAPMIIHGIKTQGARQKFSS	2,081	2,114	37	1,331	34	HLA-DRB1*04:01, HLA-DPB1*05:01
TQGARQKFSSLYISQFIIM	2,105	2,123	38	22	19	HLA-DPB1*05:01
ISQFIIMYSLDGKKWQTY	2,117	2,134	39	27	18	HLA-DPB1*05:01
SCSMPLGMESKAISDAQ	2,192	2,208	40	2	17	HLA-DPB1*05:01
MESKAISDAQITASSYFTNMFATWSPSKA	2,199	2,227	41	48	29	HLA-DPB1*04:01
TNMFATWSPSKA	2,216	2,227	42	18	12	HLA-DPB1*04:01
FATWSPSKARLHLQGRSN	2,219	2,236	43	4	18	HLA-DQB1*03:01
GRSNAWRPQVNNPKEWLQVDFQKTMKVT	2,233	2,260	44	203	28	HLA-DRB1*04:01, HLA-DPB1*05:01
GRSNAWRPQVNNPKEWLQVDFQKTMKVTG	2,233	2,261	45	205	29	HLA-DRB1*03:01, HLA-DPB1*04:01, HLA-DPB1*05:01
GVTTQGVKSLLTSMYVKEFLISSSQDGHQWTLFF	2,261	2,294	46	51	34	HLA-DPB1*04:01, HLA-DPB1*05:01
YVKEFLISSSQDGHQWT	2,275	2,291	47	2	17	HLA-DRB1*04:01

Consensus sequences are underlined and defined as the series of amino acids that are shared by all peptides belonging to the nested sets. Nested sets of at least two overlapping peptides are created. Data shown are pooled from three technical replicates.

**Table 4:** Exemplary presentation of the individual peptides mapped for human coagulation factor FVIII (UniProt accession P00451) for HLA-II alleles DRB1*15:01 (3.1-3.3) and DRB1*04:01 (4.1-4.3) that are included in the nested set for peptides sharing the consensus sequence “AEVEDNIMVTFRNQASRPYSF”.

Sample no.	Peptide																					Core no.
3.1			V	E	D	N	I	M	V	T	F	R	N	Q	A	S	R	P	Y	S	F	25
	A	E	V	E	D	N	I	M	V	T	F	R	N	Q	A	S	R	P	Y	S		
				E	D	N	I	M	V	T	F	R	N	Q	A	S	R	P	Y			
			V	E	D	N	I	M	V	T	F	R	N	Q	A	S	R	P	Y			
			V	E	D	N	I	M	V	T	F	R	N	Q	A	S	R	P	Y			
			V	E	D	N	I	M	V	T	F	R	N	Q	A	S	R	P	Y	S		
			V	E	D	N	I	M	V	T	F	R	N	Q	A	S	R	P	Y	S		
			V	E	D	N	I	M	V	T	F	R	N	Q	A	S	R	P	Y	S		
			V	E	D	N	I	M	V	T	F	R	N	Q	A	S	R	P	Y	S		
3.2			V	E	D	N	I	M	V	T	F	R	N	Q	A	S	R	P	Y	S		
			V	E	D	N	I	M	V	T	F	R	N	Q	A	S	R	P	Y	S		
3.3			V	E	D	N	I	M	V	T	F	R	N	Q	A	S	R	P	Y	S	F	
			V	E	D	N	I	M	V	T	F	R	N	Q	A	S	R	P	Y			
			V	E	D	N	I	M	V	T	F	R	N	Q	A	S	R	P	Y			
4.1			V	E	D	N	I	M	V	T	F	R	N	Q	A	S	R	P	Y	S	F	
			V	E	D	N	I	M	V	T	F	R	N	Q	A	S	R	P	Y	S		
			V	E	D	N	I	M	V	T	F	R	N	Q	A	S	R	P	Y	S		
			V	E	D	N	I	M	V	T	F	R	N	Q	A	S	R	P	Y	S		
4.2			V	E	D	N	I	M	V	T	F	R	N	Q	A	S	R	P	Y	S		
4.3			V	E	D	N	I	M	V	T	F	R	N	Q	A	S	R	P	Y	S	F	
			V	E	D	N	I	M	V	T	F	R	N	Q	A	S	R	P	Y	S	F	

Nested sets are created of at least two overlapping peptides. Sample no. represents the peptides mapped for this set for each technical replicate.

### 3.3 High coverage of previously published T-cell epitopes and our FASTMAP datasets

To compare our HLA- and antigen-specific T-cell epitope mapping pipeline FASTMAP with published data, we have chosen to test five model antigens with a high number of previously published epitopes. HLA alleles were selected regarding a high abundance and coverage of the world’s population. The final combinations of HLA type and antigen co-expressed by the Expi293F cells were selected considering HLA-type predispositions for the antigen-related disease, epitope data that were already published including well-known epitopes, and, especially for SARS-CoV-2-related proteins, the focus on HLA-I alleles and an immune response facilitated by CD8+ T cells. The BDD-rFVIII as model antigen represents a special case because previous publications by Diego et al. (37) and Jankowski et al. (38) provided detailed data on mapped epitopes for BDD-rFVIII gained with the ProPresent Antigen Presentation Assay by ProImmune (39–41).

All datasets comprising our mapped peptides clustered into cores and consensus sequences were compared to T-cell epitopes and MHC ligands and the appropriate antigen listed in IEDB. The presence of the peptide in our dataset and in IEDB is indicated by “yes”, the case that just parts of the peptide are listed is named “in parts” or “in parts (consensus yes)” in case the identified consensus sequence was covered, and no match between the peptide lists is indicated by “no”. In addition to the list of peptides exported from IEDB for both SARS-CoV-2 proteins, we compared them to a list of published COVID-19 T-cell epitopes provided by Immudex and based on several SARS-CoV-2-related publications (42–59). The mapped peptides of BDD-rFVIII were additionally compared to the epitope data published by Diego et al. (37). To include the high potential of *in silico* prediction tools and facilitate an overall comparison of our data, we included the prediction results for all antigen/HLA combinations by NetMHCpan-4.1 (for HLA-I) and NetMHCIIpan-4.0 (for HLA-II) (34, 35). For epitope prediction results, we listed the %Rank_EL for the underlined consensus sequence, the BindLevel classified into noB for no binder, WB for weak binder, and SB for strong binder, and the predicted binding affinity in nM. All data are provided in table format in the supplement (**Supplementary Table 1**).

In brief, we confirmed peptides mapped for HLA-A*02:01 in combination with BDD-rFVIII with epitopes already mapped and listed in IEDB. All our mapped peptides were predicted as a weak binder (WB) or a strong binder (SB) by NetMHCpan-4.1. The peptides mapped for the eight HLA-II alleles were almost all found in IEDB. Furthermore, we were able to reproduce almost all peptides in the dataset of Diego et al. The results of the epitope prediction went in line with the database comparison except for the DP alleles. Here, we mapped lots of peptides present in all datasets but predicted to be no binder according to the artificial neural network. The comparison of AChRα-related peptides presented on MHC-I demonstrated 100% overlap with epitopes listed in IEDB and additionally a predicted bind level of all peptides as WB and SB. IEDB results for MHC-II peptides fell in line with that, but the prediction identified our peptides as no binders. The high number of peptides mapped for SARS-CoV-2 spike glycoprotein, especially for HLA-A*11:01, was predicted to facilitate as WB or SB, although only half of the mapped peptides were described in the selected list of published COVID-19 T-cell epitopes. Comparing our mapped peptides to the ones listed in IEDB, we were able to cover all our peptides for HLA-I and -II alleles within the list of previous studies. For peptides mapped for MHC-II presentation, we were able to identify two out of four as WB and SB using the NetMHCIIpan-4.0 prediction software. The peptide data mapped for HLA-I and -II alleles in combination with SARS-CoV-2 nucleoprotein displayed a high number of shared epitopes between the alleles as well as a high sequence coverage of the antigen in general. Almost half of the peptides we have found for HLA-I alleles were published in relation to COVID-19, and IEDB provided epitope data that matched with our identified consensus sequences. Important observations were that, first, the peptides mapped were quite long in terms of the number of amino acids, and second, they were published in the past to be DR loci related. Possible explanations will be discussed in the next section. The peptides mapped for MBP showed quite the same characteristics as described for the nucleoprotein. However, in contrast to the nucleoprotein, almost all peptides identified for MBP were already mapped by other groups in the past and listed in IEDB. The *in silico* prediction forecasted almost all mapped peptides to be no binders.

To outline the key observations of the comparison of our mapped peptides for the five model antigens with IEDB, selected literature, and prediction software, we concluded that we gain a high coverage of previously published T-cell epitopes and our datasets particularly for BDD-rFVIII, AChRα, and SARS-CoV-2 spike glycoprotein. The prediction tools represent a powerful instrument to predict MHC ligands and potential T-cell epitopes but are limited to be over- or sometimes even underpredictive compared to endogenously processed MHC-bound peptides.

### 3.4 Demonstration of the importance of HLA specificity in mapping T-cell epitopes

**Figure 3:**
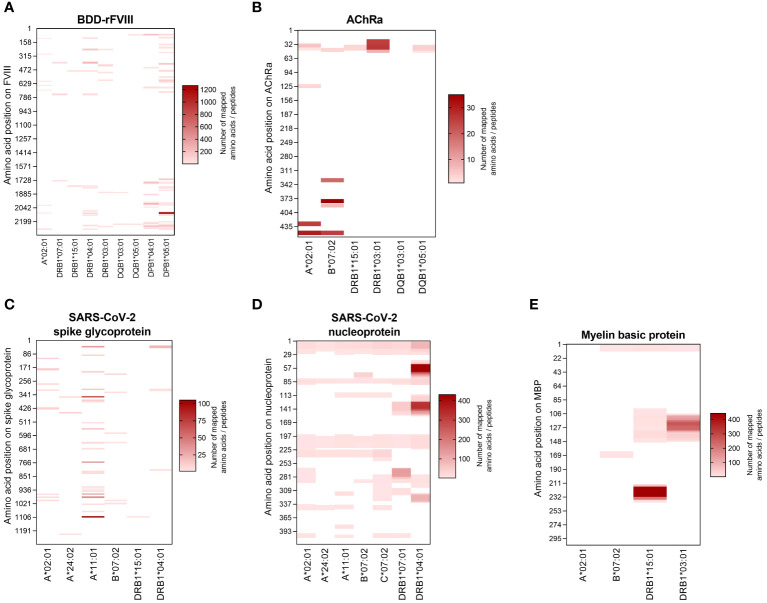
Heatmaps visualizing the HLA-specific peptide signature of the selected model antigens. The peptides mapped for each individual HLA allele are visualized according to their amino acid position on the antigen (vertical axis). The results are shown for the antigens **(A)** BDD-rFVIII, **(B)** AChRα, **(C)** SARS-CoV-2 spike glycoprotein, **(D)** SARS-CoV-2 nucleoprotein, and **(E)** myelin basic protein. The color indicates the absolute number of amino acids/peptides mapped. Data shown are pooled from three technical replicates.

**Figure 4:**
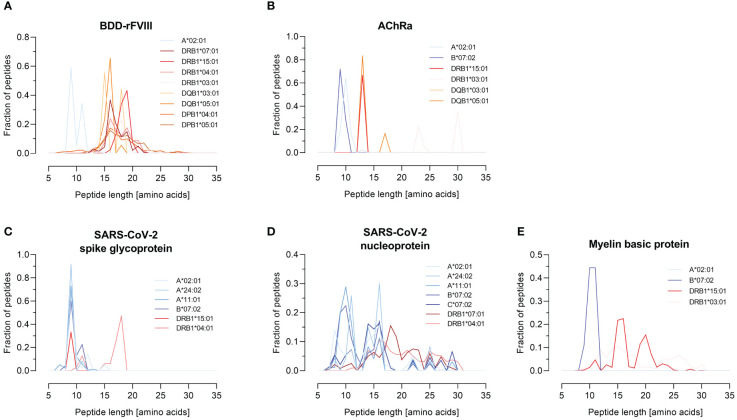
Peptide length distribution of various HLA-I and -II alleles for selected model antigens. The fraction of identified peptides for the model antigens FVIII (B-domain deleted), AChRα, SARS-CoV-2-related spike glycoprotein and nucleoprotein, and MBP is plotted against their length between 7 and 35 amino acids. The results are shown for the antigens **(A)** BDD-rFVIII, **(B)** AChRα, **(C)** SARS-CoV-2 spike glycoprotein, **(D)** SARS-CoV-2 nucleoprotein, and **(E)** myelin basic protein. Blue color spectra represent HLA-I alleles, and red color spectra denote HLA-II alleles. Data shown represent the mean of three technical replicates.

HLA specificity is critical for T-cell epitope mapping considering HLA’s polymorphic and polygenic nature and the resulting diversity in the human population. We selected various HLA/antigen combinations to evaluate our system and found an individual peptide signature of each HLA type. The individual characteristics of peptide binding are displayed in **Figure 3** showing a heatmap of each antigen combined with a varying single HLA allele. In addition, we plotted the peptide length distribution of each HLA/antigen combination shown in **Figure 4**.

BDD-rFVIII was combined with HLA-A*02:01 and eight DR-, DQ-, and DP-related HLA-II alleles. Most of the peptides were mapped specifically for a single allele; nevertheless, some parts of the protein seem to have a higher antigenic potential than others and epitopes are shared by multiple alleles. The highest numbers of peptides in terms of core sequences were mapped for the HLA-DP haplotypes. Also considering the length of the peptides mapped for BDD-rFVIII, HLA-I-related peptides consisted of 9 to 14 amino acids, and the length of HLA-II peptides varied between 12 and 34 amino acids. For AChRα, we mapped peptides for HLA-A*02:01 and B*07:02, as well as for DRB1*15:01, DRB1*03:01, DQB1*03:01, and DQB1*05:01. Again, we could see an individual peptide signature especially for both HLA-I alleles, as well as parts of the protein sequence that were presented by almost all the alleles such as the area around amino acid position 25 and 50. Analyzing the peptide length, we showed again a typical distribution of shorter HLA-I- and longer HLA-II-related peptides. For peptide mapping of the model antigen SARS-CoV-2 spike glycoprotein, we focused on presentation of MHC-I molecules represented by HLA-A*02:01, A*24:02, A*11:01, and B*07:02. Additionally, we included the HLA-II alleles HLA-DRB1*15:01 and DRB1*04:01. The heatmap showed a very individual peptide presentation for each allele, and most of the mapped peptides were presented by HLA-I. We have chosen the same approach for SARS-CoV-2 nucleoprotein adding HLA-C*07:02 for presentation on MHC-I. The peptide mapping for SARS-CoV-2 nucleoprotein resulted in a higher sequence coverage of the peptides, mainly at the N-terminus and in the central part of the protein. Aside from that, the heatmaps revealed some putatively shared epitopes of HLA-I as well as HLA-II peptides, still representing an individual peptide signature of the HLA alleles. The length of the mapped peptides mainly ranged from 14 to 30 amino acids for HLA-II-related peptides. The majority of the HLA-I peptides were distributed in the range between 8 and 12 amino acids. However, some atypically long peptides should not be ignored here and will be discussed later. For MBP, we have analyzed HLA-A*02:01, B*07:02, DRB1*15:01, and DRB1*03:01. Again, the N-terminal part of the antigen was identifiable in almost all samples except for A*02:01. The peptide length distribution revealed an average peptide length for HLA-I of approximately 10 amino acids and for HLA-II of approximately 15 and 30 amino acids.

In conclusion, these results of our FASTMAP HLA- and antigen-specific peptide mapping approach demonstrate the importance of HLA specificity in T-cell epitope mapping to display the differences in HLA-specific peptide signatures as well as the identification of shared epitopes.

## 4 Discussion

Knowledge of T-cell epitopes promises great benefits for a broad spectrum of future epitope-based therapeutics for tolerance induction in the context of autoimmune diseases as well as for several infectious diseases and cancer. Various approaches including nanoparticles coated with MHC-I or MHC-II molecules binding disease-relevant peptide fragments associated with an antigen related to autoimmune diseases like type 1 diabetes, experimental autoimmune encephalomyeltits (EAE), autoimmune hepatitis, primary sclerosing cholangitis, and collagen-induced arthritis provided first steps in the treatment of organ-specific autoimmunity without impairing normal immunity (60–66). The efficiency of epitope-based therapeutics for specific immunotherapy was also demonstrated by using antigen-processing T-cell epitopes, so-called apitopes, as tolerogenic peptides in the context of Graves disease, multiple sclerosis, and immune tolerance induction to FVIII in hemophilia A (67–69). Additionally, the MHC/peptide interaction and knowledge of the relevant T-cell epitopes is crucial for recombinant protein and drug development considering the immunosafety aspects of therapeutics.

The commonly used platforms to identify MHC binders/T-cell epitopes include systematic screenings of pools of overlapping peptides to evaluate T-cell responses, *in silico* prediction based on various algorithms as well as deep learning to identify candidate peptides, and immunopeptidomics using donor PBMCs or HLA-expressing cell lines (70–82). Challenges of peptide libraries and *in silico* tools often encompass the synthetic nature of peptides while immunopeptidomic approaches identify naturally processed peptides that are limited to the donor’s HLA genotype or the need of high cell numbers (70). Our FASTMAP immunopeptidomic-based pipeline overcomes these challenges by using a recombinant system that provides HLA and antigen specificity in combination with short processing time, high-throughput potential, and high flexibility facilitated by the transient co-expression of HLA and antigen. FASTMAP allowed us to cover a high spectrum of MHC-bound peptides identified in previous studies for many HLA alleles in combination with several model antigens.

Using this approach, we have been able to identify HLA-bound peptides for mono-allelic expression of several high-abundant HLA-II alleles covering DR, DQ, and DP haplotypes in combination with BDD-rFVIII that could be verified by previous experiments listed in IEDB as well as in a study performed to evaluate the immunogenic potential of therapeutic FVIII proteins for hemophilia A patients (37). For AChRα, the antigen for myasthenia gravis, we were able to map peptides that were listed in IEDB and were predicted to be binders for the appropriate MHC allele by NetMHCpan-4.1 and NetMHCIIpan-4.0. All our mapped MBP peptides were covered by experiments collected in IEDB, and many epitopes were identified as binders by *in silico* prediction using NetMHCpan-4.1 and NetMHCIIpan-4.0. We identified a high number of peptides related to SARS-CoV-2 spike glycoprotein that were covered by IEDB, the vast majority being predicted to be a strong or weak binder of the corresponding HLA allele. Ultimately, this is also supported by the fact that SARS-CoV-2 antigens have been intensively researched in recent years with a large number of studies performed to identify T-cell epitopes, at least to design an effective vaccine. The nucleoprotein turned out to be a unique antigen in our study. We identified many peptides, leading to almost the entire antigen sequence being mapped on our system. Many of the nucleoprotein peptides were also found in the negative control, which mapped the entire workflow only for the expression of the respective antigen and did not include HLA co-expression. In particular, hydrophobic regions of the protein were prone to produce false-positive peptides. After the removal of these false-positive peptides, a significantly reduced selection remained. We were able to make the same observation in the case of MBP. For all other antigens, we could not find any contaminating peptides in the negative control. Based on these results, we recommend that the antigen should be analyzed in advance with particular attention to hydrophobic regions of the protein sequence and, thus, potential false-positive peptides.

In contrast to previous studies using a mono-allelic approach for immunopeptidomics, we focused on the transient overexpression of a single antigen and a single HLA allele to develop a fast and reliable system with high-throughput potential allowing scalability of each step in the protocol, beginning with the potential of transfection, co-expression of HLA and antigen, and cell lysis in a deep-well format, over automated purification and washing, up to the standardized LC-MS/MS platform. These criteria to establish our FASTMAP pipeline could be fulfilled by using the Expi293F cell line as a high-yield transient expression system and the capability to process and present MHC-I- and MHC-II-bound peptides. While we have successfully demonstrated a proof of concept using the Expi293F cell line as the foundation for our aAPC system, it is important to address potential limitations that arise from the origin of Expi293F cells as non-professional APCs. Previous studies mainly focused on using B-cell lines lacking MHC-I expression (K562 and B721.221) or with reduced HLA-I expression such as C1R for the stable expression of a single HLA allele (24, 25, 81, 83, 84) considering the origin of these B-cell lines as professional APCs. Expi293F cells endogenously express HLA-I and HLA-II molecules on their surface, which may compete for peptide binding with the recombinant expressed MHC molecules despite their overexpression. To reduce the influence of the endogenous HLAs, the system could be further developed, for example, by a knock-out of the endogenous MHCs using CRISPR, to obtain a cleaner system. It would also be conceivable to identify a kind of “background” by analyzing the immunopeptidome of the endogenous HLAs compared to the recombinant ones (with and without expression of the antigen of interest). However, it should be noted here that this would be done methodically via isolation of the MHCs using appropriate antibodies and subsequent peptide elution. In addition to the issue of endogenously expressed HLAs, an analysis of the murine immunopeptidome, facilitated by a comparative analysis of two distinct strains of mice, indicated a pronounced strain-dependent influence on the immunopeptidome leading to the assumption that the immunopeptidome can be variable between different cell lines considering the factors influencing antigen processing and presentation mentioned before. Additionally, it was revealed that the immunopeptidome is specific for the expressed MHC allele and its corresponding peptide-binding motif (85). Moreover, it was reported that different cell types showed variations in peptide cleavage signatures due to the existence of a variable set of proteases and immunoproteasomal subunits resulting in individually refined versions of binding motifs (71, 86, 87). The overexpression of two components of the MHC processing and presentation pathway simplifies the very dynamic and kinetically complex intracellular pathway. Antigen expression levels greatly impact their presentation. High levels of antigen increase the likelihood of a peptide being generated and presented, and highly immunogenic antigens are more likely to be presented and later recognized by T cells. Another important point considers the antigen processing pathway influencing the antigen presentation. The efficiency of proteasomal degradation, the equipment of the cell with different chaperones and aminopeptidases influencing the peptide trimming, and the transport into the ER and loading onto MHC molecules vary based on the antigen’s structure and cellular localization (88–90). Our antigens are all equipped with a LAMP1 sig/tail sequence targeting the lysosome for efficient MHC-II presentation. Additional experiments could include an efficient MHC-I targeting to increase the number of peptides mapped for HLA-I alleles. The overexpression of distinct MHC molecules in our FASTMAP approach intended to increase the chances of effective antigen presentation, in addition to facilitating an HLA-specific peptide mapping to overcome the difficulties of HLA polymorphism within the human population. Certain HLA alleles are associated with increased susceptibility to specific diseases—others are associated with resistance—and we aimed to cover specific HLA/antigen combinations to address this predisposition (7, 17, 91, 92). However, FASTMAP has to be considered as an artificial system especially useful for drug development and not focusing on representing a system for antigen presentation in a physiological setting. The MHC-bound peptides mapped with our FASTMAP pipeline need further experiments for immunogenicity evaluation. Nevertheless, our results indicate the Expi293F cell line as a reasonable basis for aAPCs with mapped epitopes overlapping with a wide spectrum of previously published data.

We demonstrated and underlined the importance of HLA specificity in immunopeptidomics by showing individual peptide signatures and the identification of shared epitopes of selected HLA-I and HLA-II alleles that are highly abundant in the world’s population. The independence of our recombinant system from the HLA genotype of a donor also opens the door to better explore underrepresented HLA alleles (70). Especially for SARS-CoV-2 infections, a lot of effort was put into the identification of T-cell epitopes as potential candidates for vaccine design (93, 94). Mono-allelic approaches were performed by infecting cell lines with the virus or by transducing the expression construct for viral SARS-CoV-2-related antigens (84). Furthermore, a lot of research is focused on the identification of tumor antigens and how they are presented as HLA-I and -II epitopes. Studies were performed analyzing tumor ligandomes to identify HLA-I and -II ligand processing by developing a mono-allelic profiling technology (24, 25). An important feature of our FASTMAP approach is the flexibility provided by the use of a transient recombinant aAPC system. It offers almost unlimited possibilities to combine an antigen and an HLA allele by offering the potential for a high-throughput screening of naturally processed MHC-bound peptides of a wide range of alleles. However, it should not be disregarded that the essentiality of MHC binding alone is insufficient for conferring immunogenicity to a molecule. In general, the majority of predicted epitopes do not exhibit immunogenicity (95–98). To finally evaluate the potential of an MHC ligand to function as a T-cell epitope, PBMCs from disease-specific donor subsets will be needed to evaluate the immunogenicity potential of the peptide. When considering future analyses and evaluation of peptides identified with FASTMAP, peptides should also be selected based on various quality attributes. Notably, the majority of peptides were consistently detected across technical replicates, with approximately 92% found in at least two out of three replicates. This observation underscores the reproducibility and robustness of our purification workflow, affirming its suitability for subsequent peptide evaluation. We recognize the importance of peptides being present in all technical replicates, as this consistency ensures reliability in their characterization as potential T-cell epitopes. Although the Expi293F cells represent a very reliable and robust cell system, it could be advantageous to incorporate biological replicates for the identification of antigen-specific peptides in order to map any highly low-abundant peptides. A certain variability between biological replicates cannot be ruled out, as the process of antigen processing and presentation is very complex and dependent on various factors.

A quality attribute for our pipeline represents the classical peptide length distribution for MHC-I and MHC-II related peptides. We could show a distribution of peptides mainly around nine amino acids for HLA-I for BDD-rFVIII, AChRα, MBP, and SARS-CoV-2 spike glycoprotein, as well as high fractions of peptides consisting of more than 12 amino acids for MHC-II. After analyzing the distribution of peptide lengths derived from SARS-CoV-2 nucleoprotein in existing literature (7, 8, 99), we observed significant differences. Notably, we discovered exceptionally long peptides, up to 32 amino acids in length, which were mapped for HLA-I alleles. It was demonstrated in previous studies that MHC complexes have preferences in the length of peptides they present (100) and that the peptide length specificity of some HLA-I alleles (HLA-A*24:02, B*35:01, and B*07:02) is very large and includes peptides up to 25 amino acids (9). Furthermore, the overexpression of the antigen and the active targeting to the MIIC compartment in our approach can influence the peptide processing and presentation. Antigen presentation and processing are mainly facilitated by the chaperones tapasin for MHC-I and HLA-DM for MHC-II (23, 101, 102). The individual MHC-I peptidome is shaped by a combination of the proteasomal peptide cleavage, by TAP-dependent transport into the ER, and by peptide trimming by ERAP (100). An additional factor that influences the immunopeptidome represents the expression of the peptide loading chaperone HLA-DM (23, 102). The presence of HLA-DM is associated with a clearer representation of the binding motifs for HLA-DR and -DQ (24). A possible explanation for this could be the decrease in surface presentation of the MHC-II peptide placeholder CLIP and thereby an increase of presented peptides in the presence of high expression levels of HLA-DM (103). Since Expi293F cells endogenously express HLA-DM in low concentrations, it could be advantageous for future applications of FASTMAP to also co-transfect HLA-DM to investigate differences in the immunopeptidome. A high expression of the chaperone leads to changes in the peptide binding motif, which could be particularly important in relation to autoimmune diseases (24, 103). Furthermore, it should be addressed that an aAPC-based system might not be the perfect solution to mimic epitope presentation in an *in vivo* context considering the *in vivo* infected cell types, possible over-representation of epitopes resulting from antigen overexpression, and aberrant peptide presentation by professional APCs such as dendritic cells at least from a quantitative point of view (104).

Additionally, it is suggested that the restriction on the length of naturally processed MHC-I-associated peptides is predominantly determined by the availability of peptides following antigen processing, rather than the binding specificity of MHC-I molecules (9). Consequently, the overexpression and high abundance of antigens could lead to an altered peptide length distribution for nucleoprotein. In addition, we must consider the fact that we have mapped a large number of nucleoprotein peptides binding non-specifically to components of the workflow, which appear to be an antigen-specific limitation of the system. To thoroughly evaluate the influence of the active MIIC targeting on peptide presentation and the characteristics of the antigens, further experiments need to be performed without any targeting as well as with targeting MHC-I processing and presentation pathway. Nevertheless, our recombinant system provides the opportunity to study processing and presentation rules of antigenic peptides evaluating the limitations through proteolytic cleavage, transport, availability, and avidity of MHC molecules.

In summary, the ideal method to identify MHC-bound peptides would integrate the advantages of different strategies for *in silico* prediction, peptides identified in immunopeptidomics approaches using PBMCs of patients, and data from high-throughput systems that are based on aAPCs (77). Our new pipeline—FASTMAP—for HLA- and antigen-specific mapping of HLA-binding peptides represents a tool for the identification of potentially immunogenic regions of an antigen of interest, and in combination with further *ex vivo* experiments, it supports the development of new tools for immunogenicity evaluation of protein therapeutics, vaccine design, and the development of epitope-based therapeutics. Furthermore, our recombinant system can be used to gain further insights into antigen processing and presentation pathways, considering, for instance, variations in the expression of factors influencing the peptide repertoire. Owing to the high-throughput potential, our system could be used to create the large amount of allele-specific peptide mapping data that are needed to train prediction tools on endogenously processed peptide ligands to improve the algorithms and reduce the rate of false-positive predictions (71). Many patients with autoimmune disease are suffering from the high unmet medical need for antigen-specific immunotherapies also considering the patient-specific variations in the HLA genotype (105, 106). To overcome this need, our FASTMAP pipeline may facilitate the way for antigen-specific immunotherapies in autoimmune diseases with known antigen(s) to contribute to develop epitope-based therapeutics in the future that mitigate autoimmune inflammation, preserve regular immune function, are disease-specific with minimized side effects, and are curative at best.

## Data availability statement

The original contributions presented in the study are publicly available. This data can be found here: MassIVE, MSV000094062.

## Ethics statement

Ethical approval was not required for the studies on humans in accordance with the local legislation and institutional requirements because only commercially available established cell lines were used.

## Author contributions

LW: Conceptualization, Data curation, Formal analysis, Investigation, Methodology, Project administration, Visualization, Writing – original draft, Writing – review & editing. LC: Formal analysis, Investigation, Methodology, Writing – original draft, Writing – review & editing. MH: Conceptualization, Supervision, Visualization, Writing – review & editing. VS: Methodology, Supervision, Visualization, Writing – review & editing. HD: Investigation, Methodology, Writing – review & editing. BV: Supervision, Writing – review & editing. PS: Supervision, Writing – review & editing. SK: Supervision, Writing – review & editing.
