# Supplementary material for: FASTMAP—a flexible and scalable immunopeptidomics pipeline for HLA- and antigen-specific T-cell epitope mapping based on artificial antigen-presenting cells
Source: Front Immunol. 2024 May 8;15:1386160. doi: 10.3389/fimmu.2024.1386160 (PMC11109385; doi:10.3389/fimmu.2024.1386160)
Supplement: Supplementary file 3 [file DataSheet_1.pdf]

Sample ID#	Sample Set#	Short Description	Description
a	Controls	Vehicle	Complete purification workflow performed with buffers without cells
b		Negative Control	Expi293 control (untransfected) cell lysate
c		Positive Control	Expi293 control (untransfected) cell lysate spiked with 10 ng MHC I-Strep HLA-A*02:01 (YLQPRTFLL) and 10 ng MHC I-Strep HLA-B*07:02 (TPRVTGGGAM)
1	FVIII - 1-3	HLA-A*02:01 & FVIII	Expi293 cell lysate, transient expression of HLA-A*02:01 & FVIII
2		HLA-DRA*01:01 + HLA-DRB1*07:01 & FVIII	Expi293 cell lysate, transient expression of HLA-DRA*01:01 + HLA-DRB1*07:01 & FVIII
3		HLA-DRA*01:01 + HLA-DRB1*15:01 & FVIII	Expi293 cell lysate, transient expression of HLA-DRA*01:01 + HLA-DRB1*15:01 & FVIII
4		HLA-DRA*01:01 + HLA-DRB1*04:01 & FVIII	Expi293 cell lysate, transient expression of HLA-DRA*01:01 + HLA-DRB1*04:01 & FVIII
5		HLA-DRA*01:01 + HLA-DRB1*03:01 & FVIII	Expi293 cell lysate, transient expression of HLA-DRA*01:01 + HLA-DRB1*03:01 & FVIII
6		HLA-DQA1*05:01 & HLA-DQB1*03:01 & FVIII	Expi293 cell lysate, transient expression of HLA-DQA1*05:01 & HLA-DQB1*03:01 & FVIII
7		HLA-DQA1*01:01 & HLA-DQB1*05:01 & FVIII	Expi293 cell lysate, transient expression of HLA-DQA1*01:01 & HLA-DQB1*05:01 & FVIII
8		HLA-DPA1*01:03 & HLA-DPB1*04:01 & FVIII	Expi293 cell lysate, transient expression of HLA-DPA1*01:03 & HLA-DPB1*04:01 & FVIII
9		HLA-DPA1*02:04 & HLA-DPB1*05:01 & FVIII	Expi293 cell lysate, transient expression of HLA-DPA1*02:04 & HLA-DPB1*05:01 & FVIII
10	MBP - 1-3	HLA-A*02:01 & MBP	Expi293 cell lysate, transient expression of HLA-A*02:01 & MBP
11		HLA-B*07:02 & MBP	Expi293 cell lysate, transient expression of HLA-B*07:02 & MBP
12		HLA-DRA*01:01 + HLA-DRB1*15:01 & MBP	Expi293 cell lysate, transient expression of HLA-DRA*01:01 + HLA-DRB1*15:01 & MBP
13		HLA-DRA*01:01 + HLA-DRB1*03:01 & MBP	Expi293 cell lysate, transient expression of HLA-DRA*01:01 + HLA-DRB1*03:01 & MBP
14	SARS-CoV-2 Spikeprotein (SP) - 1-3	HLA-A*02:01 & SARS-CoV-2 SP	Expi293 cell lysate, transient expression of HLA-A*02:01 & SARS-CoV-2 Spikeprotein (SP)
15		HLA-A*24:02 & SARS-CoV-2 SP	Expi293 cell lysate, transient expression of HLA-A*24:02 & SARS-CoV-2 Spikeprotein (SP)
16		HLA-A*11:01 & SARS-CoV-2 SP	Expi293 cell lysate, transient expression of HLA-A*11:01 & SARS-CoV-2 Spikeprotein (SP)
17		HLA-B*07:02 & SARS-CoV-2 SP	Expi293 cell lysate, transient expression of HLA-B*07:02 & SARS-CoV-2 Spikeprotein (SP)
18		HLA-DRA*01:01 + HLA-DRB1*15:01 & SARS-CoV-2 SP	Expi293 cell lysate, transient expression of HLA-DRA*01:01 + HLA-DRB1*15:01 & SARS-CoV-2 Spikeprotein (SP)
19		HLA-DRA*01:01 + HLA-DRB1*04:01 & SARS-CoV-2 SP	Expi293 cell lysate, transient expression of HLA-DRA*01:01 + HLA-DRB1*04:01 & SARS-CoV-2 Spikeprotein (SP)
20	SARS-CoV-2 Nucleoprotein (NP) - 1-3	HLA-A*02:01 & SARS-CoV-2 NP	Expi293 cell lysate, transient expression of HLA-A*02:01 & SARS-CoV-2 Nucleoprotein (NP)
21		HLA-A*24:02 & SARS-CoV-2 NP	Expi293 cell lysate, transient expression of HLA-A*24:02 & SARS-CoV-2 Nucleoprotein (NP)
22		HLA-A*11:01 & SARS-CoV-2 NP	Expi293 cell lysate, transient expression of HLA-A*11:01 & SARS-CoV-2 Nucleoprotein (NP)
23		HLA-B*07:02 & SARS-CoV-2 NP	Expi293 cell lysate, transient expression of HLA-B*07:02 & SARS-CoV-2 Nucleoprotein (NP)
24		HLA-C*07:02 & SARS-CoV-2 NP	Expi293 cell lysate, transient expression of HLA-C*07:02 & SARS-CoV-2 Nucleoprotein (NP)
25		HLA-DRA*01:01 + HLA-DRB1*07:01 & SARS-CoV-2 NP	Expi293 cell lysate, transient expression of HLA-DRA*01:01 + HLA-DRB1*07:01 & SARS-CoV-2 Nucleoprotein (NP)
26		HLA-DRA*01:01 + HLA-DRB1*04:01 & SARS-CoV-2 NP	Expi293 cell lysate, transient expression of HLA-DRA*01:01 + HLA-DRB1*04:01 & SARS-CoV-2 Nucleoprotein (NP)
27	AChRα - 1-3	HLA-A*02:01 & AChRα	Expi293 cell lysate, transient expression of HLA-A*02:01 & AChRα
28		HLA-B*07:02 & AChRα	Expi293 cell lysate, transient expression of HLA-B*07:02 & AChRα
29		HLA-DRA*01:01 + HLA-DRB1*15:01 & AChRα	Expi293 cell lysate, transient expression of HLA-DRA*01:01 + HLA-DRB1*15:01 & AChRα
30		HLA-DRA*01:01 + HLA-DRB1*03:01 & AChRα	Expi293 cell lysate, transient expression of HLA-DRA*01:01 + HLA-DRB1*03:01 & AChRα
31		HLA-DQA1*05:01 & HLA-DQB1*03:01 & AChRα	Expi293 cell lysate, transient expression of HLA-DQA1*05:01 & HLA-DQB1*03:01 & AChRα
32		HLA-DQA1*01:01 & HLA-DQB1*05:01 & AChRα	Expi293 cell lysate, transient expression of HLA-DQA1*01:01 & HLA-DQB1*05:01 & AChRα
63	Controls - Antigens & no HLA - 1-3	Empty HLA vector & MBP	Expi293 cell lysate, transient expression of empty HLA vector & MBP
64		Empty HLA vector & SARS-CoV-2 SP	Expi293 cell lysate, transient expression of empty HLA vector & SARS-CoV-2 Spikeprotein (SP)
65		Empty HLA vector & SARS-CoV-2 NP	Expi293 cell lysate, transient expression of empty HLA vector & SARS-CoV-2 Nucleoprotein (NP)
67		Empty HLA vector & FVIII	Expi293 cell lysate, transient expression of empty HLA vector & FVIII
68		Empty HLA vector & AChRα	Expi293 cell lysate, transient expression of empty HLA vector & AChRα
